# Soluble epoxide hydrolase deficiency attenuates airway inflammation in COPD via IRE1α/JNK/AP-1 signaling pathway

**DOI:** 10.1186/s12950-023-00361-y

**Published:** 2023-11-01

**Authors:** Yue Yu, Ailin Yang, Xin He, Bo Wu, Yanjun Wu, Yunxiao Li, Shan Nie, Bo Xu, Haoyan Wang, Ganggang Yu

**Affiliations:** Department of Respiratory Medicine, Beijing Friendship Hospital, Capital Medical University, No, 95 Yong An Road, Xichen District, Beijing, 100050 China

**Keywords:** Soluble epoxide hydrolase, Endoplasmic reticulum stress, Inflammation, Cigarette smoke, COPD

## Abstract

**Background:**

Soluble Epoxide Hydrolase (sEH) metabolizes anti-inflammatory epoxyeicosatrienoic acids and critically affects airway inflammation in chronic obstructive pulmonary disease (COPD). Considering the excessive endoplasmic reticulum stress is associated with the earlier onset of COPD. The role of sEH and endoplasmic reticulum stress in the pathogenesis of COPD remains unknown.

**Method:**

16 weeks of cigarette-exposed mice were used to detect the relationship between sEH and endoplasmic reticulum stress in COPD. Human epithelial cells were used in vitro to determine the regulation mechanism of sEH in endoplasmic reticulum stress induced by cigarette smoke.

**Results:**

sEH deficiency helps reduce emphysema formation after smoke exposure by alleviating endoplasmic reticulum stress response. sEH deficiency effectively reverses the upregulation of phosphorylation IRE1α and JNK and the nuclear expression of AP-1, alleviating the secretion of inflammatory factors induced by cigarette smoke extract. Furthermore, the treatment with endoplasmic reticulum stress and IRE1α inhibitor downregulated cigarette smoke extract-induced sEH expression and the secretion of inflammatory factors.

**Conclusion:**

sEH probably alleviates airway inflammatory response and endoplasmic reticulum stress via the IRE1α/JNK/AP-1 pathway, which might attenuate lung injury caused by long-term smoking and provide a new pharmacological target for preventing and treating COPD.

## Introduction

Chronic obstructive pulmonary disease (COPD) is a heterogeneous respiratory disease characterized by continual airflow limitation and emphysema, leading to obstructive ventilatory defects [1]. Currently, it is the third-leading cause of death worldwide [2]. Cigarette smoke (CS) is the most critical risk factor, leading to a chronic inflammatory response in the lungs and causing alterations in the pathophysiology of the lungs in COPD [3]. Although the characteristics of inflammation caused by CS have been investigated for decades, there is limited knowledge regarding the mechanisms that ultimately trigger and drive these processes.

Emerging evidence indicates that endoplasmic reticulum (ER) stress may partially regulate the progression of COPD. Disturbances in protein synthesis, secretion, or disruption may lead to an accumulation of unfolded or misfolded proteins within the ER, disrupting normal ER function. This disturbance of ER homeostasis is denoted as ER stress and plays a critical role in cell viability and function [4, 5]. Previous studies displayed that the accumulation of misfolded and unfolded proteins in epithelial cells triggers ER stress and reinforces downstream pro-inflammatory and pro-apoptotic signaling through the unfolded protein response (UPR) [6, 7]. Our previous studies also indicated that cigarette smoke extract (CSE) time-dependently activated protein kinase-like endoplasmic reticulum kinase/C/EBP-homologous protein (PERK/CHOP) signaling in airway cells and induced airway epithelial cell apoptosis [8].

Moreover, ER stress inhibitor, 4-phenylbutyric acid, attenuates CS-induced emphysema and alleviates airway inflammatory responses in mice by reducing the activation of nuclear factor-κB [9, 10]. The findings suggest that airway ER stress activation participates in COPD airway inflammation through downstream inflammatory pathways. Therefore, it is logical to speculate that inhibition of CS-induced ER stress may be a novel therapeutic strategy for preventing and improving emphysema and inflammatory responses.

Soluble epoxide hydrolase (sEH) is a cytosolic enzyme mainly localized in the ER and peroxisomes. It is encoded by the gene epoxide hydrolase 2 (*Ephx2*), with N-terminal lipid phosphatase and C-terminal epoxide hydrolase activities [11, 12]. sEH broadly expression in bronchi, parenchyma, pulmonary vessels and macrophages, and the distribution is depend the oxygen-blood circulation in lung [12]. sEH hydrolyzes anti-inflammatory epoxy fatty acids to the corresponding pro-inflammatory fatty acid diols [11, 13]. sEH pharmacological inhibitors and genetic disruption stabilize epoxy fatty acids by blocking their switching [14]. sEH inhibition exerts beneficial effects in distinct disease models [15–17]. Recently, an increasing number of investigations have identified that the sEH enzyme was involved in the physiological regulation of the breathing system. In a mouse model, inhibition of sEH could prevent bleomycin-induced pulmonary fibrosis [18] and mitigate lipopolysaccharide-induced acute lung injury in mice [19]. Moreover, sEH is a physiological regulator of ER stress. Inhibition of sEH reduced high-fat diet and carbon tetrachloride-induced ER stress in adipose tissue and liver [11, 20]. A phase I clinical trial recently identified that sEH inhibitor-GSK2256294 could ameliorate smoking-induced endothelial dysfunction. It suggested that inhibition of sEH has promising therapeutic applications in COPD. However, the specific mechanism underlying regulating the airway inflammatory response in COPD by sEH remains unclear.

In our research, we utilized a mouse model of CS-induced COPD to investigate the possible role of sEH in regulating smoking-induced respiratory inflammation and emphysema. We subsequently explored the interaction of sEH and ER stress in CSE-induced inflammatory factor secretion in BEAS-2B cells.

## Materials and methods

### Reagents and antibodies

Antibodies against glucose-regulated protein 78 (GRP78; 3177S), inositol-requiring enzyme 1α (IRE1α; 3294S), c-Jun N-terminal kinase (JNK; 9252S), phospho-JNK (p-JNK; Thr^183^/Tyr^185^; 4668S), p38 (8690 S), p-p38 (Thr^180^/Tyr^182^; 4511S), extracellular regulated protein kinases (ERK1/2; 4695S) and p-ERK1/2 (Thr^202^/Tyr^204^; 4370S) were purchased from Cell Signaling Technology (Beverly, MA, USA). Antibodies against activator protein 1 (AP-1; A11378) and sEH (A1885) were purchased from ABclonal Inc. (Wuhan, China). Antibodies against p-IRE1α (AF7150) and p-PERK (DF7576) were purchased from Affinity Biosciences (Cincinnati, OH, USA). Antibodies against PERK (24390-1-AP), activating transcription factor 6 (ATF6; 66563-1-Ig), and β-actin (66009-1-Ig), as well as the goat anti-mouse and rabbit secondary antibodies (SA00001-2, SA00001-1), were procured from Proteintech Group, Inc. (Chicago, IL, USA). Goat Anti-Rabbit IgG H&L (Alexa Fluor® 488; ab150077) was purchased from Abcam (Cambridge, UK). Prime Script™ RT Master Mix Kit (RR036A) was procured from TaKaRa (Dalian, China). Tribromoethanol (T48402) and TRIzol reagent (T9424) were procured from Sigma-Aldrich (St. Louis, MO, USA). Lipofectamine™ RNAiMAX and bicinchoninic acid assay kits were purchased from Thermo Fisher Scientific (Waltham, MA, USA). IL-1β, monocyte chemoattractant protein-1 (MCP-1), IL-6, IL-8, and TNF-α enzyme-linked immunosorbent assay (ELISA) MAX™ Deluxe Kits were purchased from BioLegend (San Diego, CA, USA). The antifade mounting medium with 4’,6-diamidino-2-phenylindole (DAPI) (P0131) and quick genotyping assay kit for mouse tail (D7283M) were purchased from Beyotime Institute of Biotechnology (Shanghai, China). Tauroursodeoxycholate (TUDCA), KIRA6 and SP600125 were purchased from MedChemExpress (Princeton, NJ, USA).

### Mice and genotypic analysis

**Fig. 1:**
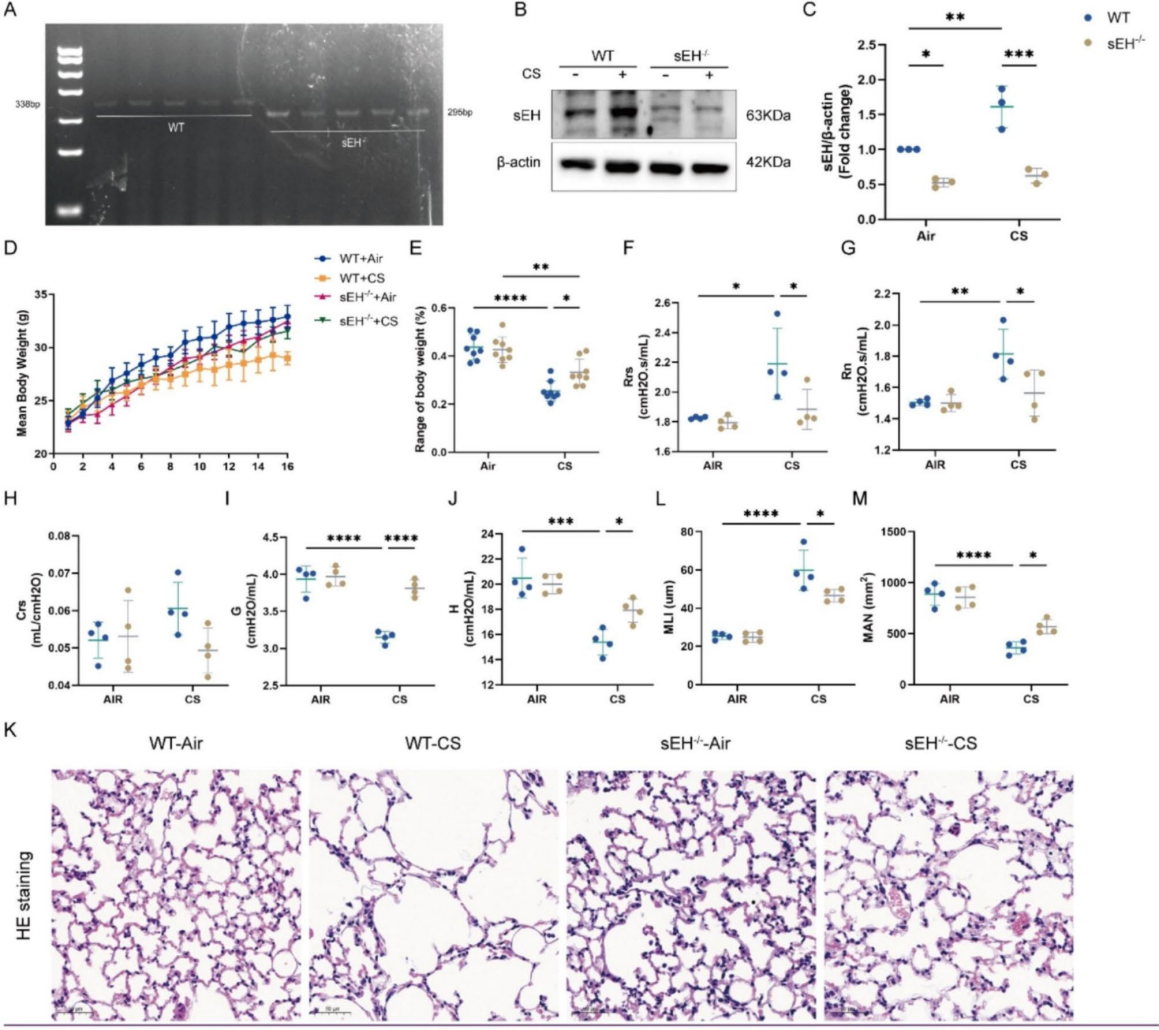
sEH deficiency mitigated changes in weight, lung function, and alveolar disruption after CS exposure. (**A**) Genotype identification result. (**B**) Western blot displaying variation in the protein expression of sEH in the lung tissue of each group. (**C**) Quantification of sEH protein expression by densitometry. (**D, E**) Changes in mouse body weight of each group after 16 weeks of exposure to air or CS; body weight was measured once a week. (**F-J**) Pulmonary function changes in each group. (**F**) Rrs and (**G**) Rn were significantly elevated; (**I**) G and (**J**) H were decreased after CS exposure. sEH deficiency inhibited Rrs and Rn, and promoted G and H. (**H**) Crs did not differ between the Air and CS groups. (**K**) Pathological manifestation in lung tissue by HE staining (×200). (**L, M**) Comparison of MLI and MAN in four groups. Data represent the mean ± SD. ^*^P < 0.05, ^**^P < 0.01, ^***^P < 0.001, ^****^P < 0.0001

Specific pathogen-free grade wild-type (WT) C57BL/6J mice (Beijing Huafukang Biotechnology Co., Ltd., Beijing, China) were used as the control group. sEH^−/−^ mice (sEH^−/−^ represents *Ephx2* gene knockout) were courtesy of the Yi Zhu Laboratory [21]. WT and sEH^−/−^ mice were maintained under standard conditions of 12-h day/night rhythm, constant temperature, and humidity levels ranging from 40 to 60%, with unrestricted access to food and water. Genomic DNA was extracted by digestion of mice tail tissues using the quick genotyping assay kit. The primers were as previously described [22]. PCR products were identified by 3% agarose gel electrophoresis, and the results were analyzed and photographed in a gel imager. A 338-base pair (bp) product was amplified for WT mice, and only a 295 bp product could be amplified for sEH^−/−^ mice (Fig. 1A). All animal studies complied with the Animal Care and Use Committee of Capital Medical University, Beijing, China.

### CS exposure

Male WT and sEH^−/−^ mice (age: 6–8 weeks, weight: 20 ± 2 g) were randomized into four groups (n = 8) and exposed to filtered air or CS. Using a fumigation device, the WT-CS and sEH^−/−^-CS groups were fully exposed to 20 cigarettes (11 mg tar, 0.8 mg nicotine/cigarette; Jiangjun, Jinan, China). Mice were exposed to CS for 2 h per day, 5 days per week, for 16 weeks at a concentration of 100–120 ppm. Animal weight was measured once a week.

### Lung function measurements

Mice were anesthetized with 2.5% tribromoethanol (0.1 ml/10 g, intraperitoneally). After anesthetization, mice were cannulated and connected to a flexiVent™ ventilator (SCIREQ, Montreal, Canada). We measured airway resistance (Rn), respiratory system resistance (Rrs), respiratory system compliance (Crs), tissue damping (G), tissue elasticity (H), and tissue hysteresivity (G/H).

### Preparation of Bronchoalveolar Lavage Fluid (BALF)

After 16 weeks of CS exposure, all mice were anesthetized and sacrificed. The trachea was dissected for tracheal intubation, and 0.7 ml of 0.9% NaCl was withdrawn using a 1 ml syringe for lung lavage; lavage was carried out thrice. The collected fluid was BALF, centrifuged at 1,000 rpm for 10 min at 4 °C. The supernatant was collected and stored at -80 °C for subsequent measurement of inflammatory factors. BALF cell precipitates were resuspended and smeared. Neutrophils were counted by Wright-Giemsa staining.

### Hematoxylin and eosin staining

Unirrigated lung tissue slices were fixed in 10% formalin for 7 days. Tissues were dehydrated in gradient ethanol and xylene, embedded in paraffin, and sliced into sections (thickness: 4 μm). Histological analysis was conducted using hematoxylin and eosin staining. The degree of emphysema was measured using the mean linear intercept (MLI) and mean alveolar number (MAN) according to previously described methodology [23].

### Transmission electron microscopy

Fresh lung tissues were fixed with electron microscope fixative at 4 °C and then fixed with 1% osmium acid for 2 h at room temperature. The tissues were dehydrated in gradient alcohol for 20 min at room temperature and then in 100% acetone twice for 15 min each time. After embedding, the embedding plates were polymerized in an oven at 60 °C for 48 h. 70 nm slices were cut using an ultra-thin sectioning machine and stained in 2% uranyl acetate saturated alcohol solution and 2.6% lead citrate solution after retrieval by copper mesh. Finally, the slices were observed under a transmission electron microscope, and images were collected for analysis.

### Preparation of CSE

A cigarette was continuously combusted using a peristaltic pump through 15 ml of phosphate-buffered saline in 3 min. The solution was adjusted to a pH of 7.2–7.4 with 1 M NaOH, and filtered through an aseptic 0.22 μm filter to remove insoluble particles. After adjusting the pH, the 100% CSE were aliquoted and stored at -80 °C [24].

### Cell culture

Human bronchial epithelial cell line (BEAS-2B) and human peripheral blood monocytes (THP-1) were acquired from the China center for type culture collection. The BEAS-2B and THP-1 cells were cultured in DMEM/F-12 or RPMI-1640 culture medium containing 10% fetal bovine serum and 1% penicillin/streptomycin in a moistened ambient with 5% CO_2_ at 37 °C. The medium was replaced every 2 days. The resuscitated BEAS-2B cells were tested between the second and fifth passages.

### sEH small interfering RNA (siRNA) preparation and transfection

The human *Ephx2* gene was silenced using siRNA. BEAS-2B cells were seeded into 12- or 6-well plates 12 h prior to transfection. *Ephx2* or negative control siRNA sequences were synthesized by RIBOBIO (Guangzhou, China). siRNA (5 nM) was transfected into BEAS-2B cells (cultured to 40–60% confluency) using Lipofectamine™ RNAiMAX Transfection Reagent according to the instructions provided by the manufacturer. Real-time quantitative PCR (qRT-PCR) analysis was used to validate *Ephx2* silencing by siRNA at 48 h after transfection.

### Transwell assay

BEAS-2B cells were incubated using the bottom chamber of a Transwell 24-well plate, and after completion of transfection or administration of pretreatment, 3% CSE was added to stimulate for 12 h. Seeded 200 µl of THP-1 cells in serum-free RPMI-1640 culture medium into the upper chamber. After 24 h, cells not crossing the pore size were gently wiped from the upper chamber, and the upper chamber was immersed in 4% paraformaldehyde for 20 min and stained in crystalline violet staining solution for 10 min. The staining was finished with three rinses using double distilled water. Finally, the migrating cell numbers were assessed under a light microscope at ×200 magnification.

### Immunofluorescence

Lung tissue sections were deparaffinized, antigen repaired, blocked with 5% goat serum, added with primary antibodies p-IRE1α, p-PERK and ATF6, and incubated overnight at 4 °C. Similarly, cell culture dishes were fixed with 4% paraformaldehyde and sealed with 5% goat serum and then added with primary antibodies p-IRE1α, p-JNK and AP-1 for overnight incubation at 4 °C. The next day, the tissue slices and cells were incubated with fluorescent secondary antibody at 37 °C for 1 h. Next, the tissue slices and cell slides were sealed using the antifade mounting medium with DAPI. Finally, the slides were observed under a confocal laser microscope (Leica).

### ELISA

The content of IL-1β and MCP-1 in serum and BALF was measured using the mouse IL-1β and MCP-1 ELISA MAX™ Deluxe kit. The concentration of IL-6, IL-8 and TNF-α in cell supernatant was assessed using the corresponding human ELISA MAX™ Deluxe kit. All operations and assays were performed according to the instructions provided by the manufacturer.

### Western blot analysis

Lung tissue and cell lysates were obtained using protease and phosphoric acid protease inhibitor-containing RIPA lysates. Protein stock solutions were separated by 8% or 10% sodium dodecyl sulfate-polyacrylamide gel electrophoresis and transferred onto polyvinylidene difluoride membranes. The membranes were incubated with primary antibodies against GRP78 (1:1,000), IRE1α (1:1,000), p-IRE1α (1:1,000), PERK (1:1,000), p-PERK (1:1,000), ATF6 (1:5,000), JNK (1:1,000), p-JNK (1:1,000), p38 (1:1,000), p-p38 (1:2,000), ERK1/2 (1:1,000), p-ERK1/2 (1:2,000), AP-1 (1:1,000), and β-actin (1:2,000) overnight at 4 °C with gentle shaking, then sequentially labeled with the corresponding secondary antibodies (1:5,000) for 2 h at room temperature. Blots were visualized using enhanced chemiluminescence.

### Real-time quantitative PCR (qRT-PCR)

Total RNA was isolated from lung tissue and cell specimens using TRIzol reagent. Reverse transcription was carried out using the Prime Script™ RT Master Mix Kit based on the instructions provided by the manufacturer. PCR analysis was conducted using the Powe SYBR Green PCR Master Mix on the 7500 Rapid Platform. The relative mRNA expression of each group was normalized to that of β-actin, and the data were analyzed using the 2^−△△CT^ approach. Primer sequences are summarized in Table 1.

**Table 1:** Primer sequences for qRT-PCR

Gene	Primer	Sequences (5’-3’)
*M-IL-1β*	Forward	TGCCACCTTTTGACAGTGATG
	Reverse	AAGGTCCACGGGAAAGACAC
*M-MCP-1*	Forward	TGATCCCAATGAGTAGGCTGGAG
	Reverse	ATGTCTGGACCCATTCCTTCTTG
*M-β-actin*	Forward	TGCTTCTAGGCGGACTGTTA
	Reverse	AACCAACTGCTGTCGCCTT
*H-IL-6*	Forward	AGACAGCCACTCACCTCTTCAG
	Reverse	TTCTGCCAGTGCCTCTTTGCTG
*H-IL-8*	Forward	ACTGAGAGTGATTGAGAGTGGAC
	Reverse	AACCCTCTGCACCCAGTTTTC
*H-TNF-α*	Forward	ACCACCACTTCGAAACCTGG
	Reverse	GTAGGCCCCAGTGAGTTCTG
*H-sEH*	Forward	GACATCGGGGCTAATCTGAAG
	Reverse	GGCTTTACTGTCACGTACCCA
*H-β-actin*	Forward	GTCATTCCAAATATGAGATGCGT
	Reverse	GCTATCACCTCCCCTGTGTG

### Statistical analysis

Data were analyzed using the GraphPad Prism 9 software (GraphPad Software, Inc., San Diego, CA, USA) and are presented as the mean ± SD. One-way or two-way analysis of variance was used for comparison among multiple groups. *P*-values < 0.05 denoted statistically significant differences.

## Results

### sEH deficiency alleviated CS-induced enlargement of airspaces and pulmonary function and weight changes

Genotypic analysis and western blot results validated the effect of sEH gene knockout and showed that CS exposure enhanced the protein expression of sEH in mice lung tissues (Fig. 1A-C). After 16 weeks of exposure to CS or air, the weight of mice in each group was significantly increased compared with that recorded at baseline (Fig. 1D). However, the weight of WT and sEH^−/−^ mice exposed to CS was reduced considerably compared with that of mice exposed to air; the degree of weight loss was more significant in WT mice (Fig. 1E). Using Flexivent to detect the lung function, Rrs and Rn increased remarkablely, and G and H decreased in the WT-CS group compared to the WT group. The sEH^−/−^-CS group showed significantly decreased Rrs and Rn, accompanied by obviously elevated G and H, but no significant changes in Crs and G/H, relative to the WT-CS group (Fig. 1F-J).

We also observed the pathological changes in lung tissue by HE staining (Fig. 1K). Compared with the WT-Air group, the WT-CS group had significantly dilated alveoli, ruptured alveolar septa, and decreased number of alveoli. These observations were accompanied by bronchial epithelial cell shedding, signs of necrosis, and extensive inflammatory cell infiltration around the bronchial wall. In the sEH^−/−^-CS group, bullae and alveolar fusion were milder than those noted in the WT-CS group. Also, the inflammatory cell infiltration was reduced. Figure 1 L and M show that alveolar disruption in mice is expressed as MLI and MAN. MLI was significantly decreased, whereas MAN was elevated in the KO-CS group compared with the WT-CS group. These results indicated that the COPD model had been successfully established. sEH deficiency markedly reduced the effects of CS exposure on body weight and pulmonary function and significantly alleviated lung pathological manifestations in mice.

### sEH deficiency suppressed airway and systemic inflammatory response after CS exposure

**Fig. 2:**
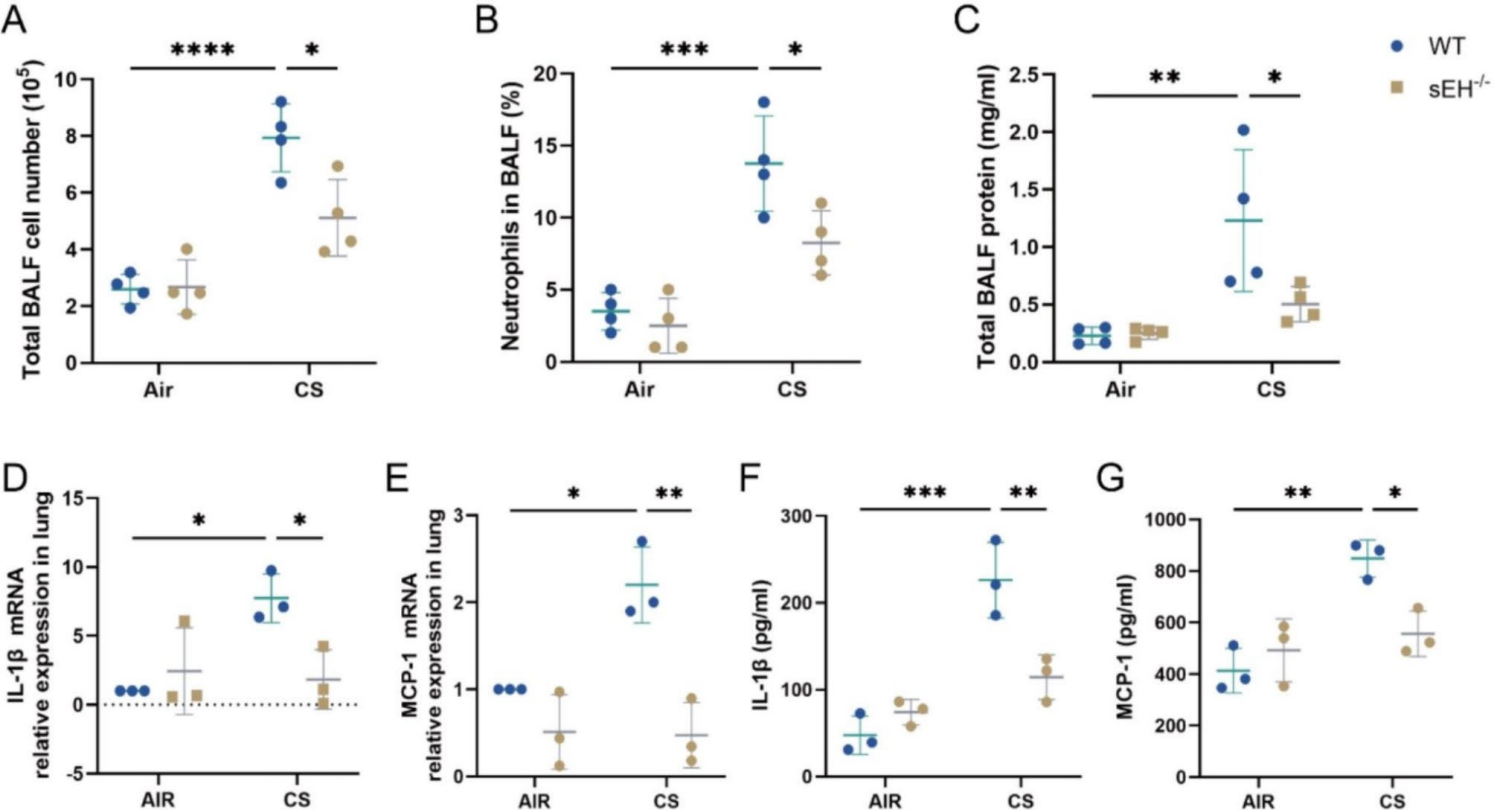
sEH deficiency attenuated CS-induced airway and systemic inflammation. (**A**) Total cell number of each group in BALF. (**B**) The percentage of neutrophils in BALF. (**C**) Total protein levels of each group in BALF. (**D, E**) qRT-PCR was utilized to assess the expression of IL-1β and MCP-1 in BALF. (**F, G**) ELISA was used to assess the expression of IL-1β and MCP-1 in lung tissue. Data represent the mean ± SD. ^*^P < 0.05, ^**^P < 0.01, ^***^P < 0.001, ^****^P < 0.0001

Airway inflammation caused by CS is closely associated with the progression of COPD. To investigate the role of sEH in CS-induced COPD inflammatory response, we measured the total protein, cell count and percentage of neutrophils in each group of BALF, as well as the expression of IL-1β and MCP-1 in BALF and lung tissue. As shown in Fig. 2A-C, in BALF, total protein, cell count and neutrophils percentage increased notably in the WT-CS group compared with the WT-Air group. Moreover, CS exposure increased the mRNA expression of IL-1β and MCP-1 in lung tissue (Fig. 2D and E). Consistently, the ELISA results revealed that CS exposure also enhanced the expression levels of IL-1β and MCP-1 in BALF (Fig. 2F and G). However, sEH deficiency markedly suppressed the release of IL-1β and MCP-1 in BALF, decreased the total cell count and neutrophils percentage in BALF, and reduced IL-1β and MCP-1 expression in lung tissue. The results showed that sEH deficiency inhibited CS-induced airway inflammatory response.

### sEH deficiency regulated CS-induced ER stress in vivo

**Fig. 3:**
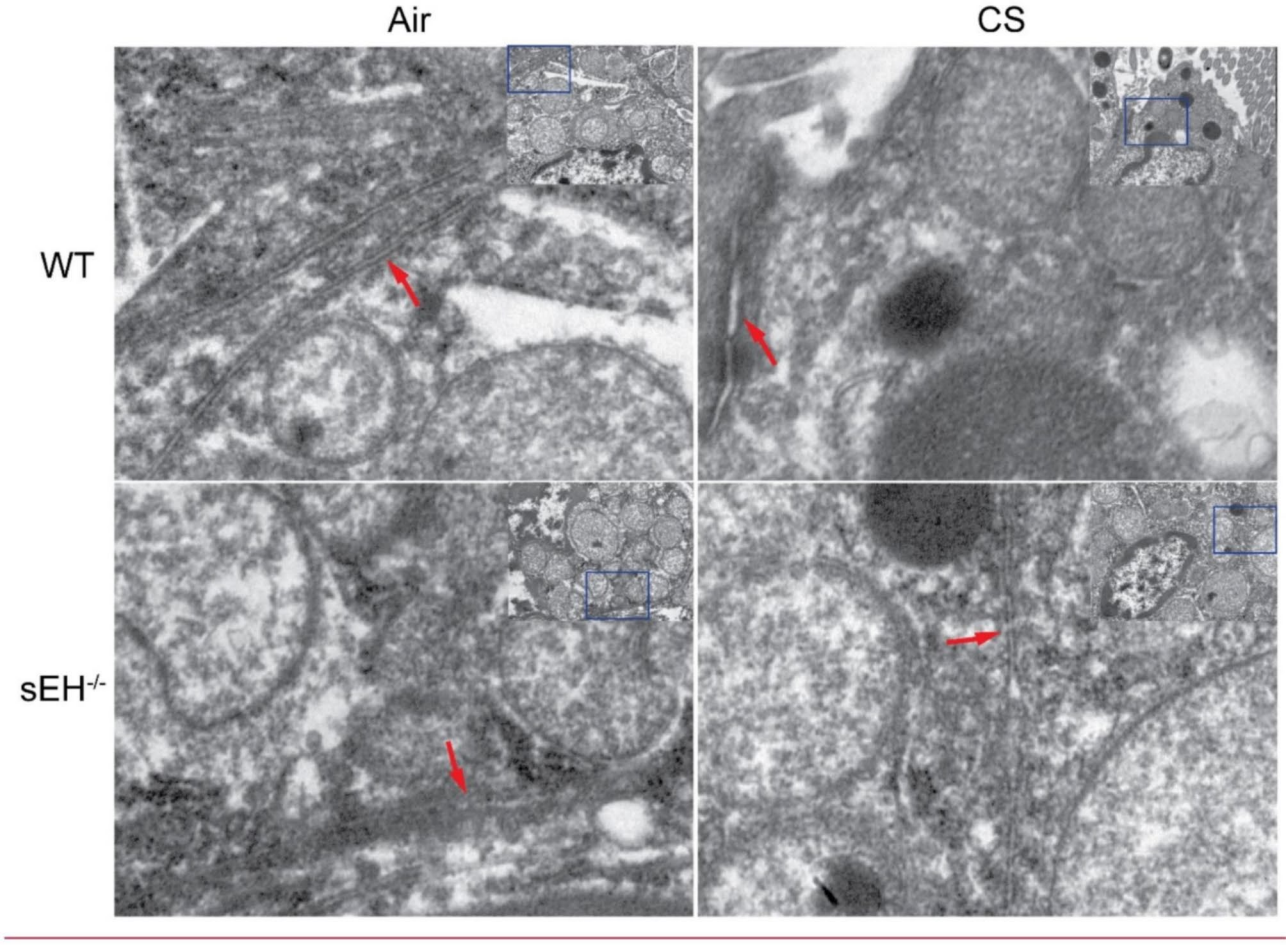
sEH deficiency attenuated ultrastructural damages in ER. TEM showed ultrafine changes in ER morphology in each group of lung tissues after 16 weeks of CS exposure. Red arrows indicate the structure of ER.

**Fig. 4:**
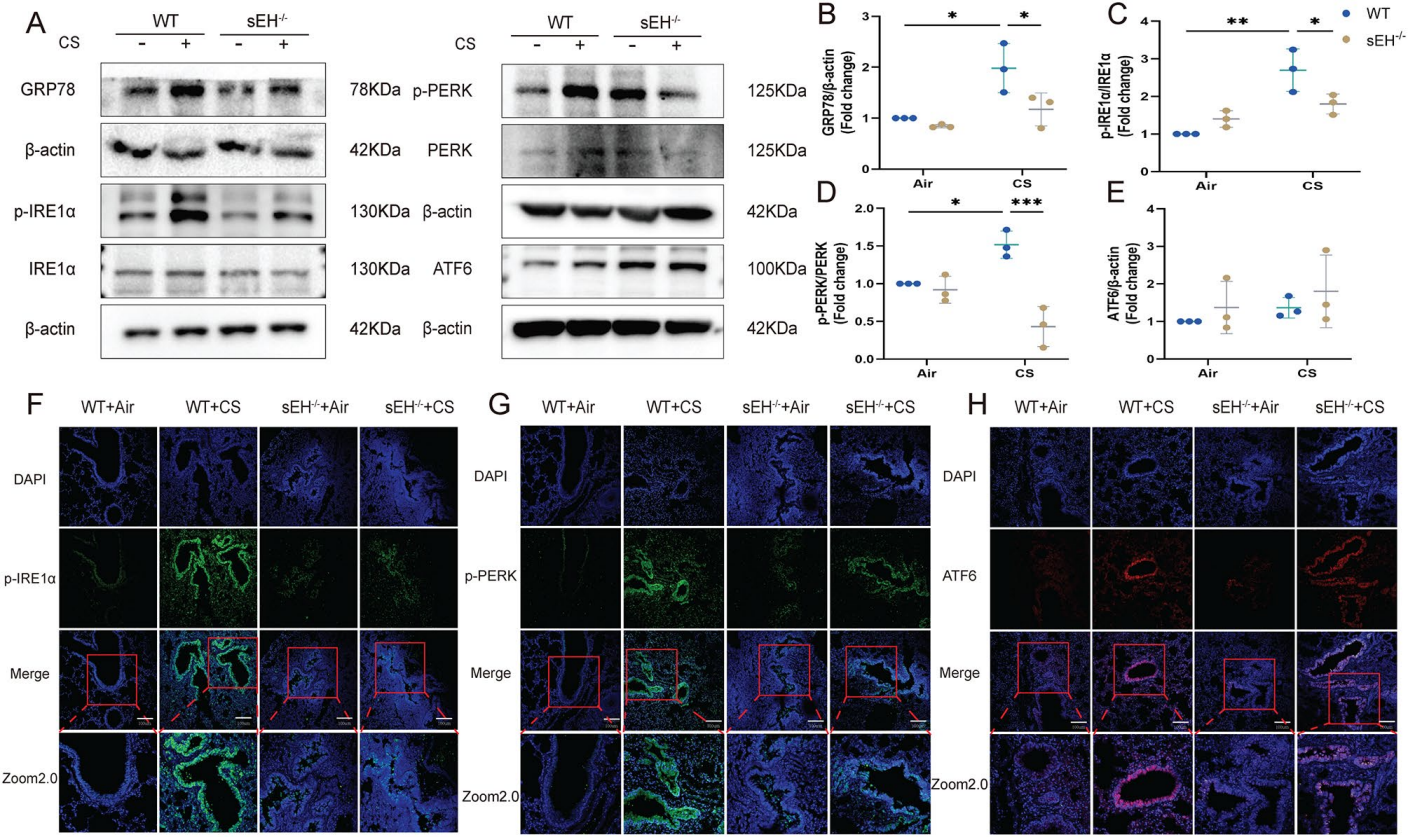
sEH deficiency suppressed CS-induced ER stress signaling in vivo. (**A**) Western blot showing changes in the protein expression of GRP78, IRE1α, p-IRE1α, PERK, p-PERK and ATF6 in the lung tissue of each group. (**B-E**) Quantifying GRP78, IRE1α, p-IRE1α, PERK, p-PERK and ATF6 protein expression by densitometry. (**F-H**) Immunofluorescence of p-IRE1α, p-PERK and ATF6 in each group’s lung alveolar/airway epithelial cells (×200). Data represent the mean ± SD. ^*^P < 0.05, ^**^P < 0.01, ^***^P < 0.001

To investigate whether sEH could influence CS-induced ER stress in lung tissue, we first detected ultrastructural changes in the ER morphology of lung tissue by transmission electron microscopy (Fig. 3). Micrographs showed visible ultrafine changes in ER morphology after CS exposure, manifested as ER swelling and dilatation. However, sEH deficiency attenuated the ultrastructural changes in ER. The present result demonstrates that sEH deficiency could prevent CS-induced ER damage. Subsequently, we determined the effects of sEH deficiency on three major signaling pathways of ER stress in lung tissue. As shown in Fig. 4A-E, CS exposure increased the protein expression of GPR78, phosphorylation of IRE1α and PERK in the lung tissue of WT mice compared with the WT group exposed only to filtered air. However, there was no significant difference in the protein expression of ATF6. Notably, the sEH^−/−^-CS group exhibited lessened ER stress than the WT-CS group, indicating that sEH deficiency attenuated ER stress. Immunofluorescence staining results further confirmed that sEH deficiency downregulated CS-induced phosphorylation of IRE1α and PERK in bronchial epithelial cells and alveolar cells, particularly reducing the activation of IRE1α in bronchial epithelial cells (Fig. 4F-H). These results manifest that CS exposure activated the IRE1α and PERK signaling pathways in lung tissue, and these effects can be diminished by sEH deficiency.

Moreover, it was shown that IRE1α activation mediates downstream factors involved in regulating inflammation. Based on these findings, we hypothesized that sEH^−/−^ might alleviate COPD airway inflammatory response by inhibiting the activation of IRE1α and its mediated downstream signaling pathway. We next tested this hypothesis in BEAS-2B cells to confirm our findings.

### sEH deficiency reduced CSE-induced inflammatory cytokine release and macrophage migration in vitro

**Fig. 5:**
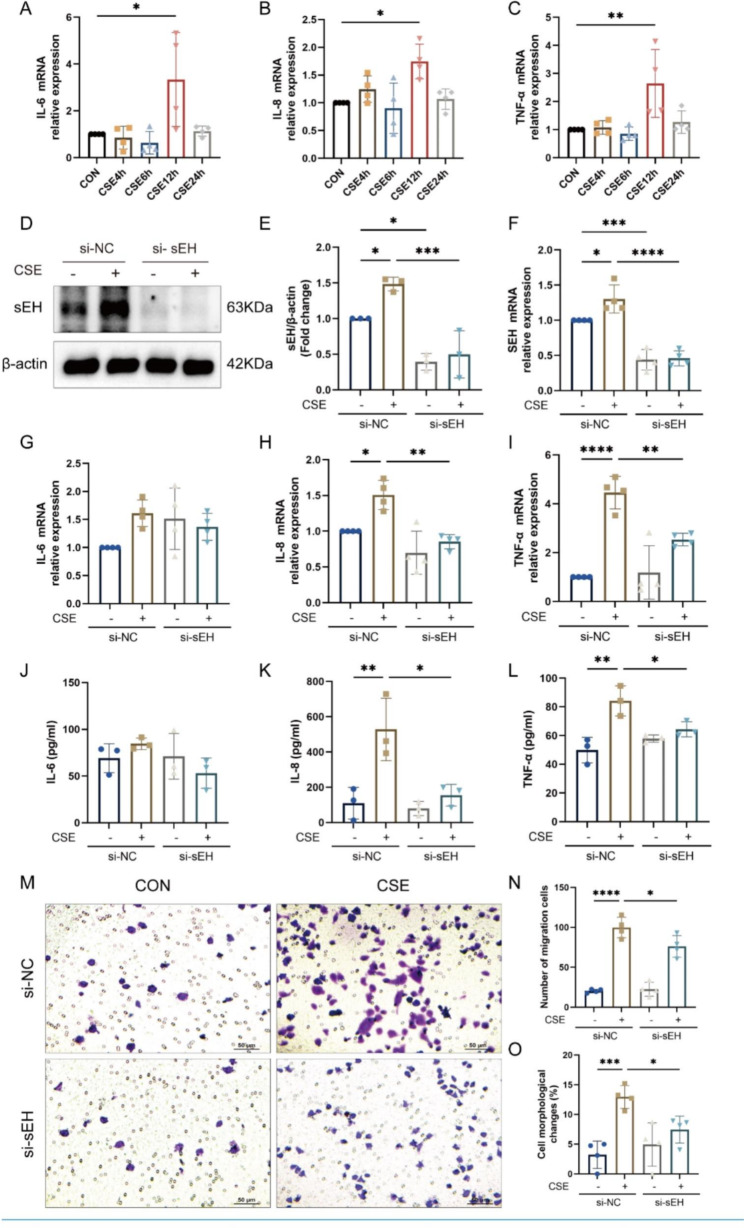
sEH deficiency inhibited the CSE-induced secretion of inflammatory cytokines and macrophage migration in BEAS-2B cells. (**A-C**) The mRNA level of the inflammatory cytokines IL-6, IL-8, and TNF-α in BEAS-2B cells after stimulation with 3% CSE for 0 (CON), 4, 6, 12, and 24 h. (**D**) Western blot was used to evaluate the protein expression of sEH in each group. (**E**) Quantification of sEH protein expression by densitometry. (**F**) qRT-PCR was used to show the changes of sEH mRNA levels in each group. (**G-I**) After transfection with si-sEH, qRT-PCR was used to assess the mRNA level of IL-6, IL-8, and TNF-α in BEAS-2B cells following stimulation with CSE for 12 h. (**J-L**) ELISA was used to evaluate the secretion of CSE-induced IL-6, IL-8, and TNF-α in the supernatant of BEAS-2B cells. (**M**) A Transwell assay was performed to detect the migration ability of THP-1. (**N**) Statistical result of THP-1 cell migration. (**O**) Statistical result of cell morphological changes. Data represent the mean ± SD. ^*^P < 0.05, ^**^P < 0.01, ^***^P < 0.001, ^****^P < 0.0001

To determine the effect of CSE stimulation on the release of inflammatory factors in BEAS-2B cells, 3% CSE was used to stimulate the cells at different time points. The qRT-PCR results showed that the mRNA levels of IL-6, IL-8, and TNF-α were increased time-dependent, and the expression reached its highest value at 12 h of stimulation (Fig. 5A-C). Therefore, this time point was selected for the following experiment. Subsequently, siRNA was used to knock down *Ephx2* (si-sEH). Western blot and qRT-PCR results verified the knockdown effect of si-sEH, and CSE stimulation elevated sEH expression in BEAS-2B cells (Fig. 5D-F). qRT-PCR and ELISA results displayed that si-sEH notably inhibited CSE-induced IL-8 and TNF-α expression. However, the differences in IL-6 expression between the groups were not statistically significant (Fig. 5G-L).

Moreover, the effects of CSE stimulation on THP-1 cell migration in each group were tested by Transwell assay. The crystalline violet staining displayed a significant increase in the number of THP-1 migrating cells and morphological change in the si-NC-CSE group compared with the si-NC-CON group (Fig. 5M-O). Compared with the si-NC-CSE group, the number of THP-1 migrating cells in the si-sEH-CSE group decreased, and some cells were polarized (Fig. 5M-O). These results suggested that sEH deficiency in bronchial epithelial cells inhibited CSE-induced inflammatory factor release and reduced CSE-induced THP-1 cell chemotaxis.

### sEH deficiency inhibited CSE-induced activation of the IRE1α/JNK/AP-1 pathway in vitro

**Fig. 6:**
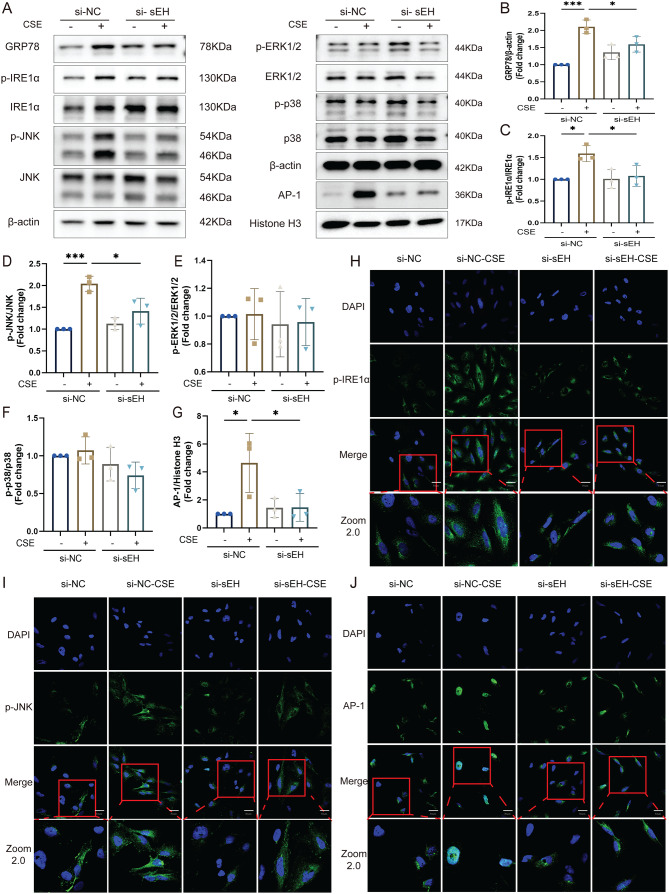
sEH deficiency inhibited CSE-induced activation of the IRE1α/JNK/AP-1 pathway in BEAS-2B cells. (**A-G**) Western blotting evaluating the effect of si-sEH on the protein expression of GRP78, IRE1α, p-IRE1α, JNK, p-JNK, p38, p-p38, ERK1/2, p-ERK1/2 and AP-1 in BEAS-2B cells after stimulation with CSE. (**H-J**) Immunofluorescence was used to determine the p-IRE1α, p-JNK and AP-1expression in each group. Data represent the mean ± SD. ^*^P < 0.05, ^***^P < 0.001

Next, we examined the expression of IRE1α and its downstream MAPK signaling pathway JNK, p38, ERK, and the transcription factor AP-1 in each group of cells. Western blot showed that CSE stimulation upregulated the expression of GRP78 and increased the phosphorylation of IRE1α and JNK in BEAS-2B cells. The expression of AP-1 was also significantly elevated in the nucleus (Fig. 6A-G). As expected, si-sEH led to a remarkable attenuation of CSE-induced ER stress and its downstream signaling. Similarly, the immunofluorescence results showed that the phosphorylated IRE1α and JNK fluorescence intensity in the cytoplasm of the si-sEH-CSE group were weakened compared with the si-NC + CSE group. The nuclear expression of AP-1 was enhanced in the si-NC-CSE group, while the intervention of si-sEH reduced the nuclear translocation of AP-1 (Fig. 6H-J). These findings suggested that sEH deficiency could inhibit CSE-induced IRE1α/JNK/AP-1 signaling pathway activation in BEAS cells.

### The interaction of sEH and ER stress in CSE-induced inflammatory factor secretion and macrophage migration in vitro

**Fig. 7:**
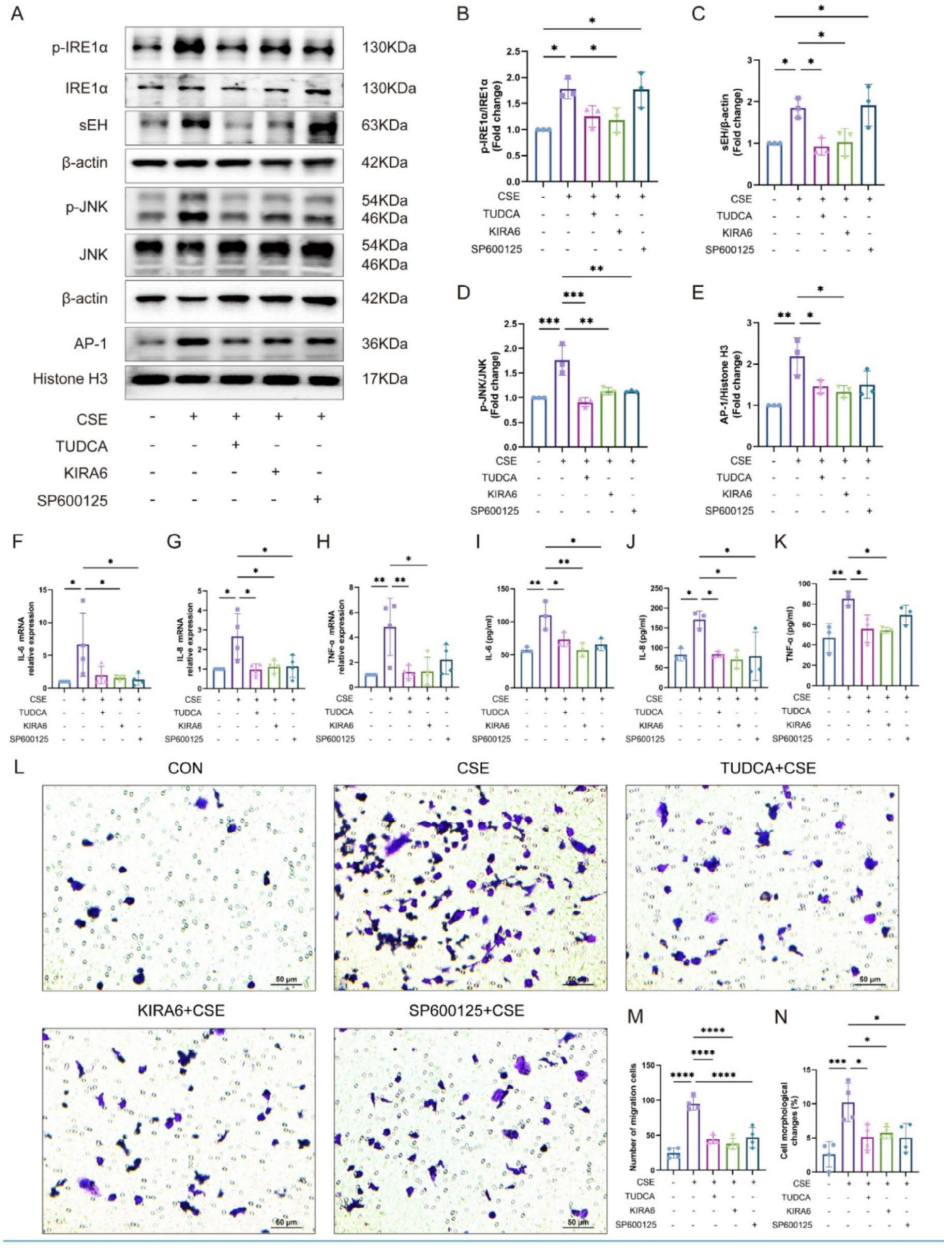
The interaction of sEH and ER stress in CSE-induced inflammatory factor secretion and macrophage migration in BEAS-2B cells. (**A-E**) Pretreatment with ERS inhibitor-TUDCA, IRE1 inhibitor- KIRA6, or JNK inhibitor-SP600125, the protein expression of IRE1α, p-IRE1α, sEH, JNK and p-JNK in BEAS-2B cells after stimulation with CSE. (**F-H**) qRT-PCR evaluating the effect of TUDCA, KIRA6, and SP600125 on the secretion of IL-6, IL-8, and TNF-α in BEAS-2B cells after stimulation with CSE. (**I-K**) ELISA analysis of the effect of TUDCA, KIRA6, and SP600125 on the secretion of IL-6, IL-8, and TNF-α in BEAS-2B cells after stimulation with CSE. (**L**) Transwell assay was performed to detect the migration ability of THP-1 in each group. (**M**) Statistical result of THP-1 cell migration. (**N**) Statistical result of cell morphological changes. Data represent the mean ± SD. ^*^P < 0.05, ^**^P < 0.01, ^***^P < 0.001, ^****^P < 0.0001

To further explore the interaction of sEH and ER stress in CSE-induced inflammatory factor secretion. We examined the effects of ERS inhibitor-TUDCA (100 µM), IRE1α inhibitor- KIRA6 (30 µM), and JNK inhibitor-SP600125 (10 µM) on CSE-induced secretion of inflammatory cytokines and sEH expression. The western blot results showed that pretreatment with TUDCA, KIRA6 and SP600125 reduced CSE-induced JNK phosphorylation and decreased nuclear expression of AP-1 compared to the CSE group (Fig. 7A-E). Furthermore, CSE-induced IRE1α phosphorylation and the expression of sEH could be inhibited by TUDCA and KIRA6 but not by SP600125 (Fig. 7A-E). qRT-PCR and ELISA results showed that TUDCA, KIRA6, and SP600125 partially reduced the CSE-induced secretion of IL-6, IL-8, and TNF-α in BEAS-2B cells (Fig. 7F-K). Transwell assay indicated that the ER stress, IRE1α, and JNK inhibitor also declined the migration number of THP-1 cells (Fig. 7L-N). Collectively, these findings suggested that the IRE1α/JNK/AP-1 pathway is involved in developing COPD airway inflammation. Inhibition of the IRE1α/JNK/AP-1 signaling pathway could alleviate the CSE-induced release of inflammatory factors from airway epithelial cells and reduce CSE-induced macrophage chemotaxis. It also indicated that sEH deficiency could ameliorate COPD airway inflammatory response by regulating the IRE1α/JNK/AP-1 signaling pathway.

## Discussion

A chronic inflammatory response to environmental irritants primarily causes COPD. sEH was recently implicated in COPD. The deficiency or pharmacological inhibition of sEH attenuates CS-induced pulmonary inflammation and emphysema [25]. However, the specific mechanism involved in this process has not been fully elucidated. Understanding the underlying regulatory mechanism may provide a molecular basis for therapies against COPD. This study observed that sEH whole-body deficiency significantly attenuated CS exposure-induced physiological and pathological manifestations and airway inflammatory response in vivo, and this therapeutic effect was closely associated with the suppression of ER stress. Similarly, the secretion of inflammatory cytokines and the migration of macrophages induced by CSE in vitro could be curtailed by administering si-sEH. Moreover, our findings demonstrated an interplay between ER stress and sEH activation in CSE-induced inflammatory response. These results suggest that inhibition of sEH and ER stress is a potential therapeutic target for COPD.

As an inflammatory disease, COPD involves innate immunity and adaptive immunity, together with the activation of structural cells, including airway cells, endothelial cells, alveolar epithelial cells, and fibroblasts [26]. Cigarettes or other stimulants could provoke airway epithelial cells and lung macrophages, releasing a variety of chemotactic mediators, especially chemokines, which attract circulating neutrophils, monocytes, and lymphocytes to the lung [27]. The present study demonstrated that prolonged systemic exposure to CS resulted in massive inflammatory cell infiltration and emphasema in lung. CS stimulate the pro-inflammation activation in both BEAS-2B cells and BALF.

Epoxyeicosatrienoic acids (EETs) exert anti-inflammatory, antioxidant, anti-apoptotic, and anti-fibrotic effects, indicating that EETs play a pivotal role in the modulation of homeostasis in the lungs [28]. However, EETs can be promptly hydrolyzed by sEH. Therefore, it is essential to restrain sEH activity to stabilize EETs and amplify their conservation effect. It has been reported that sEH deficiency or pharmacological inhibition could be therapeutic by increasing the EETs/diols ratio to reduce stressor-induced lung histopathological damage and decrease oxidative stress and inflammatory responses [25, 29]. The present study showed that sEH deficiency considerably mitigated CS exposure induced lung tissue damage and inflammatory activation. Also, in vitro, sEH deletion also reduce the release of pro-inflammation factors in BEAE-2B cells. These results suggest that sEH deficiency might significantly alleviate airway inflammation in COPD. Chemokines IL-8 and MCP-1, which are synthesized and released in large quantities in airway epithelial cells, are effective activators and chemokines of neutrophils, monocytes and have received particular attention in the pathogenesis of COPD and chronic pulmonary inflammatory diseases [30, 31]. Macrophage recruitment to the lung is one of the most crucial processes involved in the airway inflammatory response in COPD, and it plays an essential role in maintaining a sterile environment in the airways as an antigen-presenting cell [32]. However, although the number of monocytes/macrophages in the airways of COPD patients is significantly increased, their phagocytosis and killing ability of apoptotic cells is decreased [32]. Our previous research has shown that sEH deficiency lowered the number of macrophages in BALF induced by CS exposure. In this study, si-sEH preconditioning reduced the CSE-induced migration number of THP-1 cells and cell morphological changes. It was revealed that macrophage depletion was protective against CS-induced lung inflammation in mice [33]. We consider that sEH deficiency might mitigate the progression of COPD airway inflammation by suppressing CSE-induced chemokine and cytokine secretion in bronchial epithelial cells and reducing monocyte/macrophage chemotaxis.

Mounting evidence supports that ER stress induction promotes the pathogenesis of COPD [34, 35]. Our previous study confirmed that CSE could induce ER stress in bronchial epithelial cells, promoting apoptosis. It is further suggested that ER stress may be related to the development of COPD. In mammalian cells, the UPR consists of three major pathways, namely IRE1α, PERK, and ATF6. These reactions preserve intracellular homeostasis by facilitating correct protein folding, destroying fold-deficient proteins, and weakening protein translation processes [5]. Apoptosis is induced if stress persists unresolved and damage is not repaired after an initial protective phase [8, 36]. Our results demonstrated that CS exposure induced ultrastructural changes in the ER morphology of lung tissue. Furthermore, CS exposure increased the expression of ER stress-related signals IRE1α and PERK in the lung tissue of mice. Notably, sEH deficiency downregulated the expression of ER stress signaling molecules. According to immunofluorescence staining, sEH deficiency reduced CS-induced expression of phosphorylated IRE1α in bronchial epithelial cells in vivo and in vitro. This result again validated that sEH deficiency or pharmacological inhibition can alleviate ER stress [11, 20]. Previous studies have shown that suppressing excessive ER stress facilitates the fading of the disease [10]. Hence, sEH deficiency might alleviate COPD airway inflammation by inhibiting ER stress.

Activation of the mitogen-activated protein kinases (MAPKs) signaling pathway during ER stress is associated with inflammation and has an essential role in UPR-mediated signal transduction [37]. MAPKs could further trigger a series of transcription factors, such as AP-1, leading to the activation of multiple inflammatory genes and initiating an inflammatory response, thus linking ER stress and inflammatory signaling [38]. The present study found that si-sEH decreased CSE stimulation-induced JNK activation and AP-1 nuclear expression. At the same time, it had no significant inhibitory effect on CSE stimulation-induced p38 and ERK expression. Previous studies have revealed that negative regulation of the MAPKs signaling pathway effectively suppresses various smoking-induced inflammatory responses, such as reactive oxygen species production, endobronchial recruitment of inflammatory cells, and release of pro-inflammatory cytokines [39]. To further confirm whether the si-sEH intervention affects COPD airway inflammation by regulating IRE1α/JNK/AP-1 expression, ER stress, IRE1α and JNK inhibitors were used to pretreat bronchial epithelial cells. The results showed that TUDCA, KIRA6 and SP600125 could differentially suppress CSE-induced IL-6, IL-8 and TNF-α secretion. The expression of IRE1α, JNK and AP-1 was detected, further demonstrating that JNK and AP-1 are the downstream signaling molecules of IRE1α. These results suggest that sEH deficiency could alleviate the CSE-induced inflammatory response by inhibiting the ER stress signaling pathway IRE1α/JNK/AP-1. Previous research has shown that sEH is a physiological regulator of ER stress. However, the latest studies indicate that sEH is also regulated by ER stress [40, 41]. Therefore, we examined the expression of sEH after the treatment of inhibitors, and we found that ER stress and IRE1α inhibitors could reduce sEH expression, but still was no change in sEH after administration of the JNK inhibitor. Therefore, the signal transduction between sEH and IRE1α is not a simple inhibitory or facilitative relationship, and there may be a positive feedback pathway. sEH and IRE1α could interact to jointly promote the activation of downstream signals and gene transcription of pro-inflammatory cytokines, playing an influential role in the CS-induced airway inflammatory response in COPD.

However, this study also has several limitations. Firstly, we have only demonstrated that sEH could alleviate the COPD airway inflammatory response through the IRE1α/JNK/AP-1 signaling pathway, but further blocking assays of the IRE1α/JNK/AP-1 signaling pathway in mice are needed to demonstrate the role of the IRE1α/JNK/AP-1 signaling pathway in attenuating the COPD airway inflammatory response. Secondly, we only evaluated the regulatory role of sEH on IRE1α and its downstream signaling pathways. The link between sEH and the PERK signaling pathway has not been sufficiently investigated. Finally, the specific mechanism of the interaction between sEH and IRE1α has not been fully discussed, and the study of this mechanism is also the direction of our future research focus.

## Conclusions

Overall, this is the first study to link sEH and ER stress in regulating inflammation in COPD. The present data demonstrate that sEH deficiency might alleviate CS-induced airway inflammation partially by inhibiting the IRE1α/JNK/AP-1 pathway. sEH may be a promising target in the treatment of COPD.

## Abbreviations

AP-1: Activator protein 1
ATF6: Activating transcription factor 6
BALF: Bronchoalveolar lavage fluid
CHOP: C/EBP-homologous protein
COPD: Chronic obstructive pulmonary disease
CS: Cigarette smoke
CSE: Cigarette smoke extract
Ephx2: Epoxide hydrolase 2
ER: Endoplasmic reticulum
GRP78: Glucose-regulated protein 78
IL-6: Interleukin-6
IRE1α: Inositol-requiring enzyme 1α
JNK: c-Jun N-terminal kinase
MAN: Mean alveolar number
MAPK: Mitogen-activated protein kinase
MCP-1: Monocyte chemoattractant protein-1
MLI: Mean linear intercept
PERK: Protein kinase-like endoplasmic reticulum kinase
sEH: Soluble epoxide hydrolase
TNF-α: Tumor necrosis factor-α
UPR: Unfolded protein response
WT: Wild-type
